# Trypanosome Infection in Cattle and Associated Vectors in Etang District of Gambella, Ethiopia

**DOI:** 10.1155/2024/5548718

**Published:** 2024-06-06

**Authors:** Negesa Tola, Akinaw Wagari, Geremew Haile Lemu, Mohamed Kedir, Haben Fesseha Gebremeskel, Isayas Asefa Kebede

**Affiliations:** ^1^ School of Veterinary Medicine Wollega University, P.O. Box 395, Nekemte, Ethiopia; ^2^ College of Veterinary Medicine and Agriculture Addis Ababa University, P.O. Box 34, Bishoftu, Ethiopia; ^3^ Bedele National Tsetse and Trypanosomosis Investigation and Control Center, Bedele, Oromia, Ethiopia; ^4^ School of Veterinary Medicine Wolaita Sodo University, P.O. Box 138, Wolaita Sodo, Ethiopia; ^5^ School of Veterinary Medicine Ambo University, P.O. Box 19, Guder, Ethiopia

## Abstract

**Background:**

Bovine trypanosomosis produces significant economic losses due to anemia, loss of body condition, and emaciation. The disease is transmitted biologically by tsetse flies and mechanically by biting flies such as *Tabanus* and *Stomoxys*. Therefore, this research is aimed at estimating the prevalence of bovine trypanosomosis and the apparent density of its vectors in the Etang Special District.

**Methods:**

A cross-sectional study was employed from November 2016 to April 2017 for the prevalence and entomological survey. Trypanosoma species were identified using buffy coat and Giemsa staining techniques. Besides, the entomological surveys were conducted using NGU, pyramidal, biconical, and monoconical traps. The vectors were identified to their genus level based on their morphological features like size, color, wing venation, and proboscis.

**Result:**

A total of 457 bovine blood samples were collected and tested, of these 16 (3.50%) animals were positive for trypanosomosis. Similarly, 13 *T. vivax* (81.25%) and 3 *T. congolense* (18.75%) were the trypanosome species detected. The prevalence in the young (2.56%) and adult (3.99%) age groups was not statistically significant (*P* > 0.05). However, there were significant (*P* < 0.05) differences in prevalence between body condition scores, where poor is 6.31%, medium is 1.30%, and good is 0%. Moreover, the difference in mean PCV values between the parasitemic (20.97) and aparasitemic (28.58) groups was statistically significant (*P* < 0.05). *Glossina* flies were not found, although 1756 *Tabanus* and 52 *Stomoxys* biting flies were gathered. Accordingly, the overall apparent density of *Glossina* flies was zero (0), with biting flies (20.54) recorded per trap per day. Moreover, the apparent density of *Tabanus* and 52 *Stomoxys* was 39.01 and 1.18, respectively.

**Conclusion:**

This study confirmed that trypanosomosis and abundant mechanical vectors continue to be problems in the study area, resulting in cattle productivity losses. As a result, strategic management and prevention methods for trypanosomosis and associated vectors should be prioritized. Further investigation of vector needs to be conducted to clear out tsetse presence.

## 1. Background

African trypanosomosis (nagana) produces significant economic losses due to anemia, body condition loss, and emaciation. Annual cattle production losses in sub-Saharan Africa were projected to be in the range of US$ 1.0-1.2 billion [1, 2]. Bovine trypanosomosis is a serious illness that is spread both cyclically and mechanically by tsetse flies and biting flies (*Tabanus* and *Stomoxys*), respectively [1–3]. Tsetse flies are hematophagous insects belonging to the phylum Arthropoda, order Diptera, family Glossinidae, and genus *Glossina* that serve as biological vectors of trypanosomosis in animals and humans. *T. congolense*, *T. vivax*, and *T. brucei* in cattle, sheep, and goats, *T. equiperdum* in horses (Dourine), and *T. evansi* in horses and camels (surra) are the most common trypanosome species affecting livestock in Ethiopia [4–6].

Nagana is common where the tsetse fly lives in Africa, between latitudes 15° N and 29° S, and covers an area of nearly 10 million square kilometers, representing 37 nations [7]. *Glossina* habitat is divided into three types: fusca (forest), palpalis (riverine), and morsitans (savannah) habitats [7, 8]. In Ethiopia, tsetse flies are found between longitudes 33° and 38° E and latitudes 5° and 12° N. The infested areas included the lowlands and river valleys of Abay (Blue Nile), Baro, Didessa, Ghibe, and Omo and five regions (Amhara, Benishangul-Gumuz, Gambella, Oromia, and Southern Nation Nationality People Region (SNNPR) [9–11].

*Glossina pallidipes*, *G*. *morsitans submorsitans*, *G*. *fuscipes*, *G. tachinoides*, and *G. longipennis* are the five species discovered in Ethiopia. *G. pallidipes* and *G. morsitans submorsitans* are associated with savanna grassland; G. *fuscipes* and G. *tachinoides* are associated with riverbanks; and G. *longipennis* is associated with dense vegetation [12, 13]. In Ethiopia, four tsetse-borne trypanosomes have been identified: *T. congolense*, *T. vivax*, and *T. brucei brucei* of animals, and *T. brucei rhodesiense* of humans (sleeping sickness). *T. vivax* is frequently found in elevations less than 2500 meters above sea level [14–16].

Trypanosomosis has both direct and indirect consequences on a country's economy. Infected livestock causes death, morbidity, and infertility as a direct effect. Another unproductive indirect consequence that affected animals is in terms of milk, meat, traction, control, and preventative expenditures [5, 17]. If proper treatment is not established, animals die in 2-3 weeks with clinical signs of anemia, fever, abortion, roughness of hair coat, decrease of body weight, and enlargement of peripheral lymph nodes [18, 19]. Trypanosomes can be detected by identifying parasites in affected animals' blood, and several procedures are available. Many field programs use wet blood films to screen cattle for infection; thick and thin smears are used to determine the species of trypanosome [20].

Even though trypanosomosis is a major disease of cattle and causes significant economic damage in several parts of the country [21–23], no research has been conducted on the epidemiology of bovine trypanosomosis and vector distribution in the Etang Special District of Gambella Regional State. For future mitigation initiatives, this study will provide baseline data on the epidemiology of bovine trypanosomosis and its vector distribution. As a result, the particular objectives of this study were to look into the prevalence of bovine trypanosomosis in the Etang Special District of Gambella Regional State, as well as to survey potential risk factors linked with the disease and to identify the distribution of vectors in the study area.

## 2. Material and Methods

### 2.1. Description of Study Area

From November 2016 to April 2017, a study was conducted in Etang Special District, Gambella Region, Southwestern Ethiopia. The district lies 831 kilometers from Addis Ababa in the western direction and has land coverage of around 1737 square kilometers. Geographically located between latitude 08° 09′ 93^″^ and longitude 34° 04′ 20^″^ and 34° 21′ 40^″^, the altitude ranges from 350 to 480 m.a.s.l, temperature ranges from 28°C to 43°C, and typical annual rainfall ranges from 1000 to 1500 mm. There are 23327 cattle, 10016 sheep, 14343 goats, and 20802 poultry in the district [24].

### 2.2. Study Population

The animals employed in this investigation were native zebu cattle (*Bos indicus*), which are typically kept under a strict husbandry system. They were free to graze during the day and held in stables at night. A blood sample was drawn at random from all age categories, including young (<3 years old), adult (>3 years old), and both sexes of animals. Their body condition ratings were graded as good, medium, or poor [5, 25, 26].

### 2.3. Study Design and Sample Size Determination

The prevalence of bovine trypanosomosis and vector dispersion was determined using a cross-sectional study design. There had been no previous research on the prevalence of bovine trypanosomosis and the identification of its vector dispersion in the study area. Thrusfield's [27] formula was used to determine the sample size for a simple random sampling technique.

(1) $$\begin{array}{c} n=Z^{2}\times \frac{P_{\exp}\left(1-P_{\exp}\right)}{d^{2}}, \end{array}$$

where *n* is the required sample size, *P*_exp_ is the expected prevalence (*P* = 50%), *d* is the desired absolute precision (5%), and *Z* is 1.96 for a 95% confidence interval.

Based on the formula given, the maximum sample size of 384 animals was calculated, but to increase precision, 457 samples were collected from four PAs. The allocations of animals for each kebele were based on livestock populations, availability of infrastructure, and disease history reports from veterinarians working in fields. Thus, from the study sites, the individual animal was selected randomly.

### 2.4. Study Methodology

#### 2.4.1. Parasitological Study

Blood samples were taken from 457 cattle using a heparinized capillary tube after puncturing the marginal ear vein with a lancet until it fills at least 3/4^th^ of volume; then, one end was sealed with crista seal. The samples were kept in an ice box with ice packs and then transported to Bedele National Tsetse and Trypanosomosis Investigation and Control Center laboratory (BNTTICC), which was then centrifuged at 12000 rpm for 5 minutes with a capillary hematocrit centrifuge. Packed cell volume (PCV) was measured using a hematocrit reader, and values less than 24% were termed anemic [28]. By utilizing a diamond pencil, the capillary tubes were broken just 1 mm below the buffy coat and expressed on a microscopic slide, combined, and coated with a 22 × 22 mm cover slip. To determine the presence of a parasite, it was examined using a light microscope with 40x objectives [2, 29]. The positive sample was prepared into thin smears that were fixed with methanol for 5 minutes, stained with Giemsa for 30 minutes, and viewed under an 100x objective microscope. The size of the kinetoplast, the position of the nucleus, and the length of the flagellum were used to differentiate trypanosome species [30].

#### 2.4.2. Entomological Survey

To estimate the apparent density of the vector, a total of 44 different types of traps (11 NGU, 11 pyramidal, 11 biconical, and 11 monoconical traps) were placed at 200-meter intervals near riverbank and wood grassland vegetation types [31]. The used traps were almost equally deployed at selected villages: 10 traps in each PAs (Basil, Ebago, and Eliya), except Achewa PAs, which had 14 traps. Variations of deployed traps in types and numbers in PAs were due to the absence of one type of trap that covers the study sites at a time. During trapping, acetone, octanol, and cow urine were distributed as attractants in open bottles placed under each trap. The altitude, latitude, longitude, and temperature of each trap position were recorded using a global positioning system (GPS) and collected after 48 hours. The caught flies were identified using a hand lens based on their morphological traits of size, color, wing venation, and proboscis at the genus level [32, 33]. The district's climatic condition was 28-43°C during the time of the survey and measured with a hand thermometer.

### 2.5. Data Analysis

The raw data was entered into a Microsoft Excel spreadsheet, and the coding, processing, and validating were completed on this sheet. For analysis, coded data was transferred to Stata statistical software version 14 (Stata Corp, 4905 Lakeway Drive, College Station, Texas, 77845, USA). The results were expressed using the descriptive statistics, the Student *t*-test, and the univariable logistic regression analysis. The prevalence of trypanosomosis was estimated by dividing the total number of infected animals by the total number of animals investigated and multiplying the result by 100. The apparent fly density was estimated using the formula *f*/*t*/*d* (fly/trap/days). The univariable logistic regression analysis was used to compare the relationship of disease with different risk factors (peasant association, age, sex, and body condition score). The Student *t*-test was used to compare the mean PCV of infected and noninfected animals, and a probability (*P*) value less than 0.05 was considered statistically significant.

## 3. Results

A total of 457 cattle were examined from four PAs, and overall, 16 (3.5%) were positive for *trypanosome infection*. The highest prevalence (6.31%) was recorded in poor body conditions and zero (0%) in good body conditions. The prevalence of trypanosome among PAs, sexes, and body conditions is presented in Table 1.

Packed cell volume (PCV) was classified as anemic (≤24%) and nonanemic (>24%–48%), and the prevalence of trypanosome between these two categories was 14/230 (6.84%) and 2/227(1.12%), respectively (Table 2).

In this study, there were only two species of trypanosome were identified, with an overall *T. vivax* highest rate of 81.25% (Table 3).

The factors considered in the univariable logistic regression analysis of the presence of trypanosomosis were sex, age, BCS, and PAs. Of these factors, only BCS was found to be significantly (*P* < 0.05) associated with trypanosomosis while sex, PAs, and age of the animals did not have a significant effect (*P* > 0.05) (Table 4).

### 3.1. Entomological Results

The entomological survey was conducted in the selected villages, according to vegetation type and population of cattle. Thus, there were 1808 biting flies caught, and there were no tsetse flies in all villages. Accordingly, the apparent density of the *Glossina* was zero, and the biting fly was 20.54 fly/trap/days (f/t/d). The apparent density of *Tabanus* and *Stomoxys* was 39.01 and 1.18, respectively (Table 5).

## 4. Discussion

According to the current study, the prevalence of bovine trypanosomosis in the Etang Special District of Gambella Region was 16 (3.5%). It was consistent with the findings published by 3.9% [29] in Yayo, Illubabor, Ethiopia, and 2.5% [34] in Ghana. However, this is lower than the previously recorded 16.93% in Horro Guduru, Ethiopia [30], and 18.1% in Abobo and Gambela district, Southwestern Ethiopia [35]. This demonstrates the impact of parasite and vector control conducted in the adjacent district (Etang) by Bedele NTTICC [35]. Moreover, the recorded prevalence might be varied due to the technique used to detect the parasites, which is less sensitive compared to the molecular approach. Another possible reason for the variations in prevalence is the variations in the uses of prophylactic treatments with trypanocidal drugs, which mask the epidemiological situation of the disease; it appears that farmers treat their animals often themselves with drugs that are freely available on the market [36].

During the examination, two trypanosome species were identified: with the highest and lowest record of *T. vivax* (81.25%) and *T. congolense* (18.75%), respectively. This is consistent with the findings of research conducted in Southwestern Hawassa, Ethiopia, *T. vivax* 62.5% and *T. congolense* 31.25% by Denbarga et al. [37], and Northern Western Ethiopia, *T. vivax* 56.55% and *T. congolense* 43.55% by Achenef and Admas [38]. *T. vivax* was more prevalent than *T. congolense*. In this study, the observed nontsetse hematophagous flies might have a significant contribution to the high prevalence of *T. vivax* than *T. congolense.*Because *T. vivax* can be transmitted mechanically by biting flies, *T. congolense* is majorly transmitted cyclically via a tsetse fly. This study's findings coincide with the report of Abebe [16] and Cherenet et al. [39], *T. vivax* reported in an apparent tsetse-free area.

When the prevalence of cattle trypanosomosis was compared between the sexes of animals, 12 (3.42%) were female and 4 (3.74%) were male. This is not statistically significant (*P* > 0.05). It is comparable to the previous findings in Bench Maji, Southwestern Ethiopia, by Tadesse et al. [40]; Metekel and Awi zones, North Ethiopia, by Mekuria and Gadisaa [41]; and Arba Minch, Ethiopia, by Teka et al. [42]. The vulnerability to infection is the same for both sexes. Because of the shortage of feed, female animals in the study area were allowed to graze together with males. Moreover, the community prioritized dairy farming over land farming.

The prevalence recorded between ages categorized was 2.56% in young and 3.99% in adults. However, it was not statistically significant (*P* >0.05). This result agrees with the previous report by Dagnachew et al. [43] and Girma et al. [36]. The adults are more affected than young animals, because adult animals travel long distances from home for food and water and contact with vectors compared to young animals [36].

The estimated prevalence of trypanosomosis-based body conditions is as follows: poor, medium, and good were 6.31%, 1.30%, and 0%, respectively. Poor animals had the highest prevalence, which was statistically significant (*P* < 0.05). This is in line with Girma et al. [36] who reported a prevalence of 10.25%, 0.3%, and 0% in poor, medium, and good body conditions, respectively. This suggests that diseases, nutritional imbalances, and management methods may have had a role in poor body condition [44].

It is known that anemia is one of the main clinical features of African animal trypanosomosis. It is usually determined by the measurement of the PCV of individual animals [45]. Assessment of PCV in the present study revealed an overall mean packed cell volume (PCV) of 20.52% and 28.75% for parasitemic and aparasitemic animals, and it was statistically significant (OR = 6.463; *P* < 0.05). This is consistent with the reported mean PCV value of 21.52% in parasitemic and 28.75% in aparasitemic animals in Didessa District of Oromia Regional State, Ethiopia, by Gamechu et al. [46]. During the experimental period, roughly 50.33% of the samples were PCV less than 24%, but more of them react negatively to trypanosome. This could be related to a recent recovery treated with trypanocidal medicines, poor diet, helminth infection, or tick-borne sickness. From the total sampled, approximately 49.67% (PCV > 24 − 48%) were found to be in the normal range but positive for the parasite. This could be linked to a recent infection. This finding agrees with a prior study conducted by Garoma [47] and Wagari et al. [48] in the East Wollega Zone of Ethiopia.

During the entomological survey, only mechanical transmitters (biting flies) were collected; however, no *Glossina* (tsetse fly) was detected. The overall *Glossina* and biting fly apparent densities obtained were zero (0) and 20.54 fly/trap/day, respectively. This finding is consistent with the most abundant biting fly (*Tabanus* and *Stomoxys*) discovered by Achenef and Admas [38] in Debre Elias District, Northwestern Ethiopia. The absence of *Glossina* flies during the survey could be attributed to the district's high ambient temperature (28–43)°C, which is unfavorable for the tsetse fly and control mechanism implemented by Bedele NTTICC in neighboring districts. If the ambient temperature surpasses >35°C, it is not conducive to tsetse activity or mobility and increases their mortality [4]. Conversely, this study revealed the existence of *T. congolense* without its vectors (*Glossina* spp.), which could be due to the movement of animals between tsetse-infested and non-tsetse-infested areas.

### 4.1. Limitations of the Study

In the current study, only the microscopic technique was performed to detect trypanosomes; consequently, two species due to the less sensitive diagnostic method were identified. The possibility of not detecting other trypanosomes was majorly dependent on the used technique and due to the short study period and manpower in the field; information on weight, breed, skin color, and occurrence of ectoparasites such as ticks on animals was not included. Moreover, other limitations of this study are that it was done in the dry season, and thus, we were not able to analyze the effect of seasonal variation on the burden of the disease and vectors. However, previous studies in the country have shown a significantly higher prevalence of trypanosomosis and tsetse density in the long rainy season than in the dry season [49, 50]. Another limitation of this study is that it does not indicate tributaries and the main river that can play a major role in the presence of tsetse flies. Cattle movement for grazing and watering is not indicated to show the presence of *T. congolense* with that of tsetse flies. The geographical picture was also important to see the neighboring tsetse-infested or free district.

## 5. Conclusion

During the investigation, the occurrence of bovine trypanosomosis was 3.50% and the apparent density of the vector was 20.54 f/t/d in four villages of the Etang Special District. Only biting flies were detected among the vectors gathered, but no *Glossina* species. The absence of tsetse flies may be because the environmental temperature was not favorable to tsetse activity during the investigation. *T. vivax* infection prevalence (81.25%) and *T. congolense* infection prevalence (18.75%) were detected. In poor body conditions and low PCV animals, the prevalence of trypanosome infection was greater. This study found that trypanosomosis and its vectors were still a danger to livestock productivity and economic losses in the district in both direct and indirect ways. As a result, multiple tactical approaches for controlling and preventing trypanosomosis and its vector should be enhanced. Further research into trypanosome infection and a vector survey should be conducted in the district.

## Figures and Tables

**Table 1:** The prevalence of cattle *trypanosomosis* in the study area.

Variables	Categories	No. of examined	No. of positive	%	95% CI
Peasant associations	Bazil	100	2	2	0.50–7.72
	Achewa	126	4	3.17	1.19–8.20
	Ebago	108	4	3.70	1.39–9.51
	Eliya	123	6	4.88	2.20–10.48

Sex	Female	350	12	3.42	1.95–5.95
	Male	107	4	3.74	1.40–9.60

Age	≤3 year	156	4	2.56	0.96–6.66
	>3 year	301	12	3.99	2.27–6.90

BCS	Poor	206	13	6.31	3.69–10.59
	Medium	230	3	1.30	0.42–3.98
	Good	21	0	0	—

CI = confidential interval; % = prevalence; No. = number.

**Table 2:** Mean PCV of parasitemic and aparasitemic animals.

PCV	No. of examined	No. of positive	%	95% CI for %	Mean	OR	95% CI for OR	*P* value
≤24%	190	13	6.84	4.00–11.45	20.97%	6.463	1.815-23.008	0.004
>24%	267	3	1.12	0.36–3.44	28.58%			
Total	457	16	3.50	2.15–5.64	24.75%			

PCV = packed cell volume; No. = number; CI = confidential interval; % = prevalence.

**Table 3:** The prevalence of *trypanosome* species in four PAs.

PAs	Total sampled	No. of positive	Spp. of trypanosome	
			*T. vivax*	*T. congolense*
Achewa	126	4	3 (75%)	1 (25%)
Ebago	108	4	3 (75%)	1 (25%)
Eliya	123	6	5 (83.33%)	1 (16.66%)
Basil	100	2	2 (100%)	0 (0%)
Total	457	16	13 (81.25%)	3 (18.75%)

Spp. = species; *T*. = *Trypanosoma*.

**Table 4:** Univariable logistic regression analysis of the association of trypanosomosis with different factors.

Variables	Categories	%	OR	95% CI	*P* value
Peasant associations	Bazil	2	ref	—	—
	Achewa	3.17	1.606	0.288-8.954	0.589
	Ebago	3.70	1.884	0.337-10.521	0.470
	Eliya	4.88	2.512	0.496-12.731	0.266

Sex	Female	3.42	ref.	—	—
	Male	3.74	1.093	0.345-3.464	0.879

Age	≤3 years	2.56	ref	—	—
	**>**3 years	3.99	1.577	0.500-4.975	0.436

BCS	Medium	1.30	ref	—	—
	Good	0	1	—	—
	Poor	6.31	5.096	1.431-18.148	0.012

OR = odds ratio; Ref = reference category; CI = confidence interval; % = prevalence.

**Table 5:** The apparent density of flies caught at the study site.

PAs	No. of traps deployed	Type of vegetation	*Glossina*	Total	Other biting flies					
					*Tab.*	f/t/d	*Stom.*	f/t/d	Total	f/t/d
Basil	10	WGL	—	—	264	26.40	6	0.60	270	13.70
Achewa	14	Baro River	—	—	535	38.21	14	1	549	19.61
Ebago	10	Gnimulu Lake	—	—	179	17.90	9	0.90	188	9.40
Eliya	10	Baro River	—	—	778	77.80	23	2.30	801	40.05
Overall	44		—	—	1756	39.01	52	1.18	1808	20.54

WGL = woody grassland; f/t/d = fly per trap per day; *Tab*. = *Tabanus*; *Stom*. = *Stomoxys*; f/t/d = fly/trap/days; PAs = peasant associations.

## Data Availability

The data sets developed and analyzed were included in this study.
